# Introducing the hormonal Intrauterine Device in Madagascar, Nigeria, and Zambia: results from a pilot study

**DOI:** 10.1186/s12978-021-01300-x

**Published:** 2022-01-06

**Authors:** Kendal Danna, Grace Jaworski, Bakoly Rahaivondrafahitra, Francia Rasoanirina, Anthony Nwala, Masauso Nqumayo, Gina Smith, Pierre Moon, Ashley Jackson, Sarah Thurston, Amanda Kalamar

**Affiliations:** 1 Population Services International (PSI), 1120 19th St NW #600, Washington, DC 20036 USA; 2 PSI Madagascar: Immeuble Arboretum-ex Village Des Jeux de la Francophonie Ankorondrano, 101 Antananarivo, Madagascar; 3 Society for Family Health (SFH) Nigeria, Justice Ifeyinwa Nzeako House, #8 Port Harcourt Crescent, Area 11, Garki, Abuja, Nigeria; 4 SFH Zambia, Plot No. 549 Ituna Rd, Ridgeway, Lusaka, Zambia; 5 WCG Cares, 12400 High Bluff Drive, Suite 600, San Diego, CA 92130 USA

**Keywords:** Hormonal IUD, Hormonal IUS, LNG-IUS, LARC, Continuation, Satisfaction, Acceptability

## Abstract

**Background:**

The hormonal Intrauterine Device (IUD) is a highly effective contraceptive option growing in popularity and availability in many countries. The hormonal IUD has been shown to have high rates of satisfaction and continuation among users in high-income countries. The study aims to understand the profiles of clients who choose the hormonal IUD in low- and middle-income countries (LMICs) and describe their continuation and satisfaction with the method after 12 months of use.

**Methods:**

A prospective longitudinal study of hormonal IUD acceptors was conducted across three countries—Madagascar, Nigeria, and Zambia—where the hormonal IUD had been introduced in a pilot setting within the of a broad mix of available methods. Women were interviewed at baseline immediately following their voluntary hormonal IUD insertion, and again 3 and 12 months following provision of the method. A descriptive analysis of user characteristics and satisfaction with the method was conducted on an analytic sample of women who completed baseline, 3-month, and 12-month follow-up questionnaires. Kaplan–Meier time-to-event models were used to estimate the cumulative probability of method continuation rates up to 12 months post-insertion.

**Results:**

Each country had a unique demographic profile of hormonal IUD users with different method-use histories. Across all three countries, women reported high rates of satisfaction with the hormonal IUD (67–100%) and high rates of continuation at the 12-month mark (82–90%).

**Conclusions:**

Rates of satisfaction and continuation among hormonal IUD users in the study suggest that expanding method choice with the hormonal IUD would provide a highly effective, long-acting method desirable to many different population segments, including those with high unmet need.

## Plain language summary

The hormonal IUD is a popular contraceptive method choice where it is available. While the method has been around for decades it remains out of reach for many populations in low- and middle-income countries. The hormonal IUD offers both highly effective protection from pregnancy as well as non-contraceptive benefits that appeal to users in the US and Europe, but there is limited evidence available to explore the acceptability and use of this method among users in lower resource settings. This research looks at the profile of users who chose to use the hormonal IUD during a pilot introduction in Madagascar, Nigeria, and Zambia. Over a period of 12 months, researchers followed up with these users to understand who continued to use the method over time and their satisfaction with the method. Findings show that the hormonal IUD was broadly appealing in these settings among users from multiple demographic segments, including among users who had not used other methods of contraception in the past. When followed-up at 3 and 12 months, satisfaction with the method was high and most users in all three countries were still using the method. The findings suggest that the hormonal IUD could be well received and may contribute to greater uptake of voluntary modern contraception in lower resource settings.

## Introduction

The hormonal Intrauterine Device (IUD) is a popular contraceptive choice where it is available [1]. The highly effective and reversible method releases a low dose of the hormone levonorgestrel into the uterus. The hormonal IUD offers unique therapeutic and non-contraceptive benefits, such as lighter bleeding and relief from menstrual pain, and reduced side effects relative to other hormonal methods [2]. In the United States, where the Mirena^®^ brand of hormonal IUD was first marketed in 2001, the method has contributed to an increase in the prevalence of all Intrauterine Devices (IUDs) among American women using contraception, from 2% of the method mix in 2000 to more than 12% in 2014 [3]. The hormonal IUD has also provided an alternative for women who may otherwise turn to a more invasive hysterectomy procedures for treatment of menorrhagia and conditions such as endometriosis [4]. Rates of hormonal IUD satisfaction and continuation are high [5–7]; however, most of the evidence generated to date has been from high-income markets.

The hormonal IUD has remained widely inaccessible for populations in low- and middle-income countries (LMICs), with product cost as a key barrier. Low volumes of commercially available hormonal IUD products, from suppliers such as Bayer and Pregna, are available in some LMIC countries. A range of costs to the client have been documented at up to $400, putting the method out of reach for the majority of the population [1]. Since 2003, the International Contraceptive Access (ICA) Foundation, a public–private partnership between Bayer Pharmaceuticals and the Population Council, has donated limited quantities of an unbranded hormonal IUD product to organizations in 36 countries that offer women free or low-cost family planning services, with roughly 21,000 units donated per year between 2017–2019 [8].

Affordability of the hormonal IUD has improved with recent shifts in the global supply landscape. In 2015, the US Food and Drug Administration approved a lower cost hormonal IUD from Medicines360, a non-profit pharmaceutical company. Medicines360 registered their product under the brand name Avibela^®^ in four African countries, as of 2020. Global health stakeholders and procurement agencies have shown increased interest in the potential to expand method choice with the hormonal IUD in LMIC markets [1].

In 2015, The United States Agency for International Development (USAID) convened the Hormonal IUD Technical Working Group. This coordination group aims to increase the range of highly effective contraceptive options available to women through a commitment to strengthen the body of evidence for the hormonal IUD. The group includes donors, suppliers, research agencies, and service delivery organizations that work together to explore and address the barriers that have, thus far, excluded the hormonal IUD from developing markets [1]. This research was designed to meet the need for stronger evidence in these contexts. In 2021, both USAID and UNFPA added the method to their commodity procurement catalogs, meaning the hormonal IUD will soon be available for widespread procurement through regular commodity procurement channels. This research will continue to inform global efforts for introduction and scaled-up access to this method.

### Introduction of the hormonal IUD into LMIC programs

From 2017 to 2020, Population Services International (PSI) introduced the hormonal IUD in three countries through two projects funded by USAID. Through the Expanding Effective Contraceptive Options (EECO) project led by WCG Cares (WCG), PSI added the hormonal IUD to voluntary family planning programs in Madagascar and Zambia. Through the Support for International Family Planning and Health Organizations 2 (SIFPO2) project, PSI did the same in Nigeria. These countries were selected for introduction with the aim of understanding the uptake and acceptability of the hormonal IUD in both public and private sectors with varying demand generation strategies, all within the context of access to a broad mix of methods. This evidence will be used to improve, and potentially further expand, global efforts to introduce the hormonal IUD.

The three programs took place in countries where a minority of women use contraception. According to Track20, the modern contraceptive prevalence rates (mCPR) in 2019 among all women are 36% in Madagascar, 14% in Nigeria, and 35% in Zambia. Method skew, which can signal that women have too few contraceptive options, is particularly evident in Madagascar, where more than 60% of contraceptive users use injectables. In Nigeria and Zambia, injectables, oral contraceptive pills, implants, and condoms are the most widely used methods. IUD use is low in all three countries, with a 2.1%, 5.2%, and 1.5% share of the method mix in Madagascar, Nigeria, and Zambia, respectively [9–11].

PSI Madagascar and the Society for Family Health (SFH) Nigeria began offering the hormonal IUD through private facilities in their social franchise networks beginning in 2018 and 2017, respectively. These social franchises comprise private clinics supported by PSI with medical training in all methods (including copper IUDs and implants) and continuing education, supportive supervision for quality assurance, medical equipment, and consumables. At these sites, clients paid a service fee to receive a voluntary hormonal IUD insertion. In contrast, SFH Zambia offered the hormonal IUD through public facilities where all methods are free. Master trainers led the provider trainings in the hormonal IUD and supervised insertion and removal procedures until providers met competency requirements.

The three programs raised community awareness of the new method within the context of informed choice. In all contexts Interpersonal Communication (IPC) agents, trained community health educators, offer counseling and education on contraceptive method benefits, limitations (including side effects), characteristics and features. IPC agents in Madagascar and Nigeria were trained and employed by PSI and SFH, respectively, spoke with individuals and groups about the range of family planning methods available, including the hormonal IUD, and made referrals to providers for balanced counseling and a wide range of family planning methods. In Nigeria, this IPC support for demand generation was programmed only at one quarter of facilities offering the hormonal IUD, meaning only a proportion of clients would have interacted with and IPC agent prior to their service. The Madagascar program also used hormonal IUD promotional materials, including clinic wall posters and brochures that highlighted the hormonal IUD as a new contraceptive method available and described the potential for lighter periods as a primary benefit of the method. In Zambia, IPC agents employed through the public sector were provided additional training and education materials by SFH Zambia, they included the hormonal IUD in their communication with communities about family planning and they also educate communities in other health areas.

To understand the introduction of the hormonal IUD into these three LMIC markets from the user perspective, PSI designed this accompanying pilot study to descriptively explore key questions related to the programmatic introduction of this new method. The primary objectives were to understand: (1) What are the demographic profiles of the clients who will use this product?; (2) Does introduction of the hormonal IUD help reach new modern contraceptive users (current non-users)?; (3) Do users of the hormonal IUD ‘switch’ from other methods and if so, from what other methods (e.g., from short-acting methods)?; (4) Do users experience a high level of satisfaction with this method?; and (5) What are continuation rates at 12 months and reasons for removal?

## Study design

To answer these questions through the pilot study, in each country, women ages 18–49 were recruited at the time of their visit to facilities after counseling and choosing the hormonal IUD. Women were eligible to participate if they were between the ages of 18 and 49, chose to receive the hormonal IUD, and consented to participate. Following their hormonal IUD provision, recruitment, and informed consent, a baseline client interview was conducted among all women who agreed to take part in the study and a mobile phone number was collected for follow-up. The research team in Madagascar and Nigeria then attempted to follow-up with all women by phone at 3- and 12-months after their baseline survey. In Zambia, follow-up was conducted in person and a member of the research team visited a woman at home.

Facilities were purposively selected in each study country, focusing on high client volume facilities that were already providing other LARC services. In all countries, facilities were selected from the regions where the hormonal IUD had been introduced but prior to the time of this introduction, pilot facilities had not been trained to offer the hormonal IUD and, and as such, historical IUD service provision was not factor in selection of facilities for this study. In Madagascar, facilities were selected from Mahajanga, Toamasina, Antsiranana, and Antananarivo provinces. In Nigeria, facilities were selected from Benue, Cross River, Enugu, Oyo, Kano, Katsina, Kaduna, Taraba, Lagos, Abia, Rivers, Akwa Ibom, Taraba, Edo, and Imo States. In Zambia, facilities from the Copperbelt and Muchinga provinces were included.

To calculate the sample size needed for analysis of the primary outcome, we use the Stata sampsi command to calculate the sample size needed to estimate the proportion of women satisfied with the hormonal IUD at 12-months with 95% confidence and 80% power. To estimate the expected proportion of women satisfied, we use data from the literature on client satisfaction with the method at follow-up. Based on previous studies regarding client satisfaction with the hormonal IUD in other settings, 75% was used as a conservative reference for satisfaction [7, 12, 13]. Using this baseline reference for client satisfaction and adjusting for intra-cluster correlation at the facility level, the intended sample size at the 12-month endline was 150 respondents in each country. The sample size needed at recruitment and baseline was inflated to account for anticipated loss to follow-up—by 30% in Madagascar, by 40% in Nigeria, and by 10% in Zambia. In all countries except for Zambia, whose follow-up procedures differed from the other two countries, loss to follow-up was much higher than anticipated. As such, the baseline sample size was increased midway through the study, proportional to the amount of follow-up experienced up to the date that was beyond the anticipated follow-up (i.e., if the loss to follow-up was anticipated to be 20% during initial sample size calculations and the experienced loss to follow-up was 30%, the baseline sample size was inflated by 10% and recruitment at baseline continued), to further account for loss to follow-up.

Client satisfaction at 12-month follow-up was assessed by asking each client who reported that she was still using the hormonal IUD she received at baseline to rate her satisfaction on a five-point Likert scale. We estimate contraceptive continuation of the method using Kaplan–Meir time-to-event probabilities to calculate the cumulative probability of contraceptive continuation at 12-months, in line with how the widely used Demographic and Health Surveys (DHS) calculate contraceptive (dis)continuation [14]. By using time-to-event probabilities, we include data for all women in the study, inclusive of those who participated at baseline but not follow-up and those that participated at 3-month follow-up but not at 12-month follow-up. All women who participated at only baseline are censored at 0 months, all women who participated at the 3-month follow-up but not the 12-month follow-up, and who did not report discontinuing the method at 3 months, are censored at 3 months. And all women who participated in the 12-month follow-up but did not report discontinuing the method are censored at 12 months. Contraceptive continuation was calculated using information gathered from all clients at follow-up, including those who reported no longer using the hormonal IUD, when they stopped using the method, and their reason for discontinuation, which we rank by frequency of report to ascertain main reasons for discontinuation. At the 3 and 12-month follow-ups, women were asked if they were still using the hormonal IUD and if not, for how many months did they use prior to stopping. For this analysis, women were classified as discontinuing their method if they reported they stopped using the hormonal IUD for reasons other than wanting to become pregnant or that they were no longer sexually active. Given that there were variations in the exact follow-up dates, any woman who stopped using the method prior to their 4th month of use was classified as discontinuing by the 3-month follow-up and classified as discontinuing by the 12-month follow-up if they discontinued prior to their 13th month. Women who switched from the hormonal IUD to another modern method within the follow-up period were considered to have discontinued the method for this analysis as they discontinued using the hormonal IUD specifically, even though they continued using family planning.

Descriptive analyses were used to assess the demographic characteristics—age, education, marital status, and parity—of the women choosing the hormonal IUD, their stated reasons (e.g., their primary reason and their secondary reasons using structured response options commonly found in the literature/DHS surveys) for choosing the hormonal IUD, information about their contraceptive use history prior to using the hormonal IUD from the baseline survey, and to assess satisfaction with the method, discontinuation, and reasons for discontinuation from the 3 and 12-month follow-up surveys. To distinguish women by the types of methods ever used, women were categorized into four mutually exclusive categories: (i) women who had never used any method, (ii) women who had only ever used traditional methods, (iii) women who had ever used short-acting methods, or (iv) women who had ever used a Long-Acting Reversible Contraceptive (LARC) (this would include women who had used both a short-acting method and/or a traditional method and a LARC in the past). These same four categories were used to group women based on the methods they had used from any point in the previous 3 months to the day of their facility visit, showing which method(s) they used before switching to the hormonal IUD.

The study was approved by each country’s local Institutional Review Board: Comité d’Ethique de la Recherche Biomédicale auprès du Ministère de la Santé in Madagascar (Approval number 082), National Health Research Ethics Committee (NHREC) in Nigeria (Approval number NHREC/01/01/2007-15/05/2017), and ERES Converge in Zambia (Reference Number 2018-Jun-028).

## Results

The analytic sample comprises participants who were interviewed at the 12-month follow-up as well as those who reported that they discontinued use of the hormonal IUD at the 3-month follow-up point and therefore were not required to participate in the 12-month follow-up interview. In Madagascar, 108 of 242 baseline respondents were included (55.4% loss to follow-up); in Nigeria, 76 of 208 baseline respondents were included (63.5% loss to follow-up); in Zambia, 155 of 166 were included (6.6% loss to follow-up).

### Demographics of hormonal IUD users

Table 1 presents the demographic profile for study respondents in each country at both baseline and for study respondents that participated in the 12-month follow-up survey. At baseline, there was generally an even distribution among the age groups of respondents, except in Nigeria, where respondents skewed older and very few were under 25. The majority of women in all three countries were married/living together with a partner and had a secondary or higher education. Most women in each country had at least one child. At 12-month follow-up, the distribution of demographic characteristics remains mostly consistent for age, education, marital status, and parity across all three countries. While there was high loss to follow-up from baseline to 12-month surveys, there was no differential or systematic loss to follow-up, with the exception of Nigeria, where women who participated in the 12-month follow-up tended to be older than the full baseline sample of women, though this difference in age distribution from baseline to follow-up is not statistically significant.

**Table 1:** Demographic profile of study respondents: hormonal IUD users in Madagacar, Nigeria, and Zambia

Variable	Madagascar^*^		Nigeria^*^		Zambia^*,a^	
	Baseline N = 242 (%)	12 month follow-up N = 108 (%)	Baseline N = 208 (%)	12 month follow-up N = 76 (%)	Baseline N = 166 (%)	12 month follow-up N = 155 (%)
Age						
18–24	73 (30.2)	28 (25.9)	12 (5.8)	1 (1.3)	28 (16.9)	27 (17.4)
25–29	51 (21.1)	24 (23.2)	42 (20.2)	16 (21.0)	35 (21.1)	33 (21.3)
30–34	46 (19.0)	27 (25)	62 (29.8)	29 (38.2)	48 (28.9)	44 (28.4)
35 or older	72 (29.8)	29 (26.9)	92 (44.2)	30 (39.5)	53 (31.9)	50 (32.3)
Pearson χ^2^		p = 0.99		p = 0.99		p = 0.99
Education						
Never attended school or did not complete primary school	18 (7.4)	9 (8.3)	6 (2.9)	0 (0)	33 (19.9)	32 (20.6)
Primary completed	25 (10.3)	10 (9.3)	16 (7.7)	8 (10.5)	45 (27.1)	42 (27.1)
Secondary or higher	199 (82.2)	89 (82.4)	186 (89.4)	68 (89.5)	87 (52.4)	80 (51.6)
Pearson χ^2^		p = 0.95		p = 0.44		p = 0.99
Marital status						
Single	45 (18.6)	13 (12.0)	3 (1.4)	0	16 (9.6)	15 (9.7)
Married/living together	190 (78.5)	92 (85.2)	198 (95.2)	75 (98.7)	140 (84.3)	130 (83.9)
Widowed/divorced/separated	7 (2.9)	3 (2.8)	7 (3.4)	1 (1.3)	9 (5.4)	9 (5.8)
Pearson χ^2^		p = 0.43		p = 0.45		p = 0.99
Parity						
* Mean* ± *SD*	1.7 ± 1.3	1.7 ± 1.3	3.3 ± 1.8	3.3 ± 1.8	3.3 ± 2.0^b^	3.2 ± 1.9
T-test		p = 0.52		p = 0.61		p = 0.99

*The analytic sample consists of those who completed baseline, 3-month follow-up, and 12-month follow-up questionnaires as well as those that reported discontinuation at the 3-month follow-up point. The demographics of the analytic sample were compared with the demographics of all respondents who were interviewed at baseline. No differential loss to follow-up was found

^a^While 166 respondents participated in the baseline and 155 respondents participated in the follow-up in Zambia, not all respondents answered all survey questions. As a result, in Zambia proportions will not always equal 100%

^b^This was calculated from the n = 150 that responded to the parity question in Zambia

### Reasons for choosing the hormonal IUD

Across study countries, desired duration of method use was a primary reason for choosing the hormonal IUD—cited by 31.5% of women in Madagascar, 69.7% of women in Nigeria, and 69.7% of women in Zambia, while its effectiveness was also important to women in Madagascar and Nigeria. The hormonal IUD had a desirable side effect profile for a third of women in Zambia and a desirable bleeding profile for nearly 1 in 5 women in Madagascar and more than a quarter of women in Zambia. Just over 36% of women in Zambia liked the method’s convenience while a recommendation from the provider was important for a quarter of Nigerian women in the study. Table 2 presents the number and percentage of women who selected each reason for choosing the hormonal IUD.

**Table 2:** Reasons for choosing hormonal IUD among hormonal IUD users in Madagascar, Nigeria and Zambia

Reasons for choosing the hormonal IUD	Madagascar N = 108 (%)	Nigeria N = 76 (%)	Zambia N = 155 (%)
It’s effective	58 (53.7)	16 (21.1)	26 (16.8)
Desired duration of use (appropriate for desire to space or limit)	34 (31.5)	53 (69.7)	108 (69.7)
Desired bleeding profile	19 (17.6)	6 (7.9)	42 (27.1)
Desired side effect profile	18 (16.7)	6 (7.9)	52 (33.6)
Belief of other positive health outcome or treatment of medical condition (including gynecological/bleeding issues)	16 (14.8)	2 (2.6)	0
Recommendation from provider	12 (12.0)	19 (25.0)	18 (11.6)
Convenient (does not require regular maintenance)	7 (6.5)	9 (11.8)	56 (36.1)
Recommendation from friend	6 (5.6)	6 (7.9)	2 (1.3)
Desired price	4 (3.7)	0	0
Protect fertility	4 (3.7)	8 (10.5)	15 (9.7)
Safe for use (sometimes considering other medical condition)	3 (2.8)	2 (2.6)	11 (7.1)
Private/discreet	3 (2.8)	1 (1.3)	47 (30.3)
Desired low hormone level	1 (0.9)	0	0
Other or not sure	1 (0.9)	0	2 (1.3)

### Prior method use and method switching

Table 3 categorizes respondents based on their contraceptive use history by type of methods they have ever used in their lifetime and by type of methods they have used recently (in the past 3 months).

**Table 3:** Contraceptive method use history among hormonal IUD users in Madagascar, Nigeria, and Zambia

Contraceptive method use history	Madagascar N = 108 (%)	Nigeria N = 76 (%)	Zambia^a^ N = 155 (%)
Contraceptive use history by prior method type			
Never used a method	7 (6.5)	6 (7.9)	31 (20.0)
Ever used only traditional methods	1 (0.9)	3 (4.0)	1 (0.7)
Ever used short-term methods	65 (60.2)	20 (26.3)	100 (64.5)
Ever used a LARC	35 (32.4)	47 (61.8)	22 (14.2)
Contraceptive use history over the past 3 months by method type			
Has not used a method in the past 3 months	33 (30.6)	24 (31.6)	40 (25.8)
Has used only traditional methods in the past 3 months	7 (6.5)	8 (10.5)	2 (1.3)
Has used short-acting methods in the past 3 months	47 (43.5)	35 (46.1)	94 (60.6)
Has used a LARC in the past 3 months	21 (19.4)	9 (11.8)	18 (11.6)

^a^While 155 respondents participated in the follow-up in Zambia, not all respondents answered all survey questions. As a result, in Zambia proportions will not always equal 100%

Across all study countries, most women reported having used a method recently, but between 6 and 20% of women had never used any method. For those who reported prior method use, most had used only short-acting methods, except for in Nigeria, where nearly two-thirds reported having used a LARC at least once before. For the 68–74% of women who had recently used a method prior to the hormonal IUD, most reported recent use of short-acting methods, while 20% or less of women in each country reported recently using a voluntary LARC.

In addition to prior contraceptive use history, women were asked at baseline what method they would have chosen to receive had the hormonal IUD not been available. Notably, in Madagascar and Nigeria, many women (46% and 29%, respectively) stated that they would have left without a modern method had they not been able to receive the hormonal IUD (Table 4). In Zambia, more than half of women (54.2%) would have opted for a short-acting method, while 20–45% of women across the study countries would have chosen a different LARC method.

**Table 4:** Alternative modern method choice if hormonal IUD was not available among hormonal IUD users

Alternative modern method choice	Madagascar N = 108 (%)	Nigeria N = 76 (%)	Zambia* N = 155 (%)
Nothing	50 (46.3)	22 (29.0)	8 (5.2)
Short acting method	32 (29.6)	18 (23.7)	84 (54.2)
LARC	23 (21.3)	34 (44.7)	57 (36.8)
Permanent	0	0	1 (0.6)
Don’t know	3 (2.78)	2 (2.6)	1 (0.6)
No response	0 (0)	0 (0)	4 (2.5)

*While 155 respondents participated in the follow-up in Zambia, not all respondents answered all survey questions. As a result, in Zambia proportions will not always equal 100%

### Satisfaction with method

At the 3 and 12-month follow-up interviews, women were asked to rank their satisfaction with the hormonal IUD on a five-point Likert Scale (very satisfied, satisfied, neutral, dissatisfied, very dissatisfied). Table 5 presents method satisfaction among women at 3-month follow-up and 12-month follow-up, collapsed into two categories, “satisfied” and “neutral or dissatisfied,” disaggregated by age, 24 years old or younger versus 25 years or older. In all three countries, the majority of all women in both age groups reported being satisfied with the method at follow-up. In Nigeria and Zambia, the percentage of participants satisfied with the method increased between the 3-month and 12-month follow-up point for both age groups, most notably among Zambia participants 24 or younger (70.4% satisfied at 3-months to 92% satisfied at 12-months among younger participants, though the difference between age groups is not statistically significant). In Madagascar, however, the percent of participants satisfied with the method decreased from 3-months to 12-months in both age groups (93.3% to 66.7% among women 24 or younger; 87.7% to 80.3% among women 25 and older, though the difference between age groups is not statistically significant).

**Table 5:** Method satisfaction among hormonal IUD users at time of last follow-up interview by age

Country	Age group	Satisfied (%)	Neutral or dissatisfied (%)
Madagascar			
3-month follow-up N = 151	24 or younger	42 (93.3)	3 (6.7)
	25+	93 (87.7)	13 (12.3)
12-month follow-up N = 95	24 or younger	16 (66.7)	8 (33.3)
	25+	57 (80.3)	14 (19.7)
Nigeria			
3-month follow-up N = 98	24 or younger	1 (100)	0
	25+	74 (76.3)	23 (23.7)
12-month follow-up N = 73	24 or younger	1 (100)	0
	25+	62 (86.1)	10 (13.9)
Zambia			
3-month follow-up N = 155	24 or younger	19 (70.4)	8 (29.6)
	25+	85 (66.9)	42 (33.1)
12-month follow-up N = 146	24 or younger	23 (92.0)	2 (8.0)
	25+	101 (83.5)	20 (16.5)

### Method continuation

**Fig. 1:**
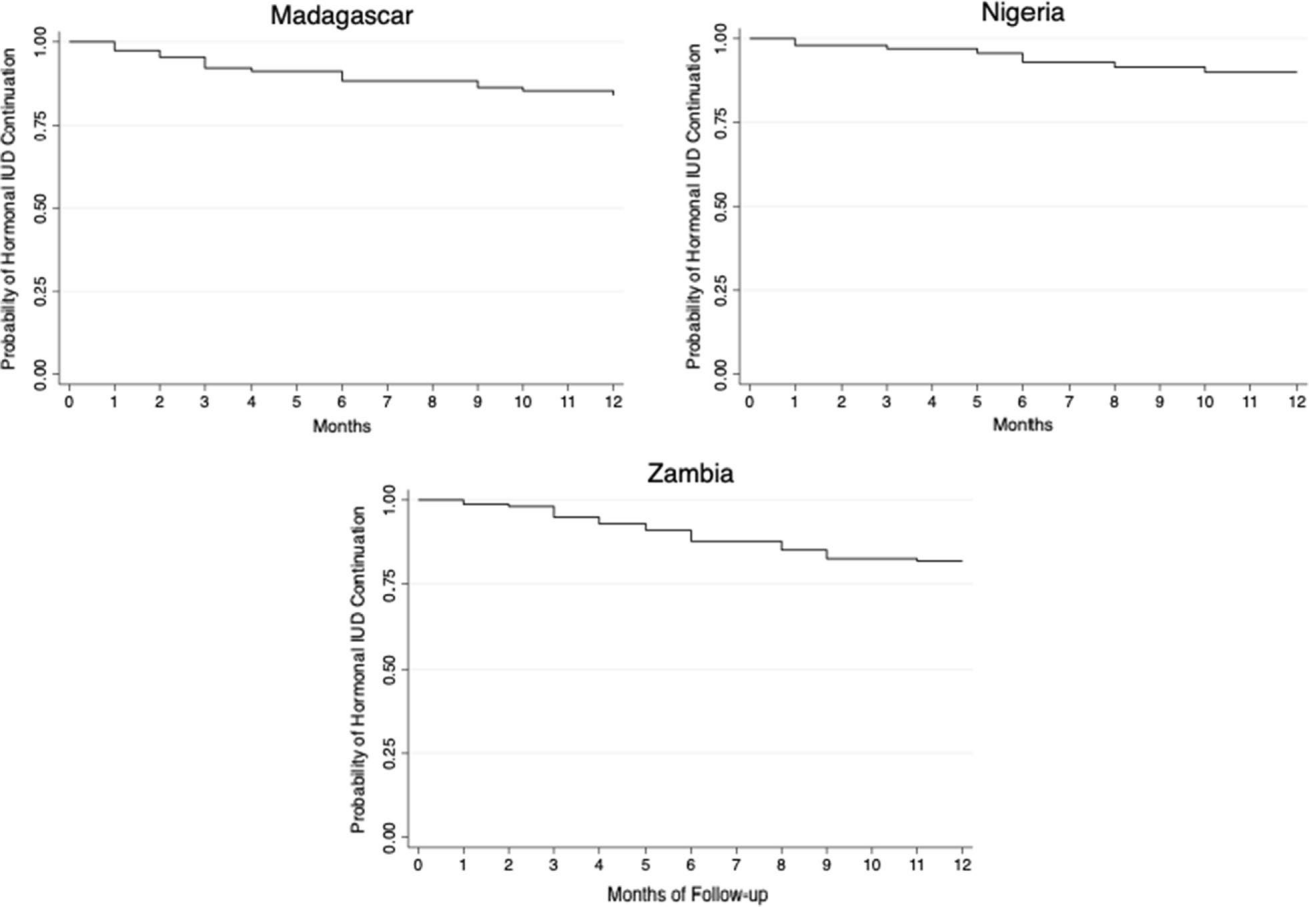
Kaplan–Meier time-to-event curves of the hormonal IUD in Madagascar, Nigeria, and Zambia

Continuation rates were constructed through survival curves using Kaplan Meier cumulative hazard models (Fig. 1). Continuation probability at 12 months in Madagascar was 84.0% (95% CI 76.0–90.0), in Nigeria was 90.0% (95% CI 80.7–94.9), and in Zambia was 81.8% (95% CI 74.7–87.1).

In Madagascar, among the 7.9% who discontinued by the 3-month follow-up, the most common reason for discontinuation was pain while using the method, while among the 10.5% who discontinued by the 12-month follow-up the most common reason for discontinuation was bleeding disturbances. In Nigeria by the 3-month follow-up (where 3.1% discontinued), the most common reason was that the method was expelled while the most common reason at the 12-month follow-up (where 8.2% discontinued) was bleeding disturbances. The most common reason for discontinuation in Zambia at the 3-month follow-up (5.8% discontinued) and the 12-month follow-up (13.7% discontinued) was bleeding disturbances. Table 6 presents a full ranking of all reasons for discontinuing use of the hormonal IUD by country.

**Table 6:** Reasons for discontinuation ranking

	Madagascar^a^		Nigeria^a^		Zambia^b^	
	Discontinued prior to 3 months follow-up n = 13 Rank #	Discontinued prior to 12 months follow-up n = 10 Rank #	Discontinued prior to 3 months follow-up n = 3 Rank #	Discontinued prior to 12 months follow-up n = 6 Rank #	Discontinued prior to 3 months follow-up n = 9 Rank #	Discontinued prior to 12 months follow-up n = 20 Rank #
Pain with method	1	2	2	2	–	4
Other reason	2	5	–	4	–	–
Partner complained/did not want me to have it	3	–	–	4	3	4
Method expelled	3	–	1		2	3
Side effects	5	4	2	2	-	2
No response	5	–	–	–	–	–
Wanted to become pregnant	–	3	–	–	3	–
Bleeding disturbances	–	1	–	1	1	1
Not having sex	–	–	–	4	–	–

Ranking by most common (rank # = 1) to least common (rank # = 5). Rank order is determined by the percentage of respondents selecting the reason for discontinuing use of the hormonal IUD by 3 months

^a^This question was fielded as a multi-response question where respondents could give multiple reasons for discontinuing their use of the hormonal IUD

^b^This question was fielded as a single response question where respondents could give only one reason for discontinuing their use of the hormonal IUD

## Discussion

In these introductory settings, the hormonal IUD provided a unique and desirable contraceptive option. Users who chose this method came from a range of demographic backgrounds and reported high rates of satisfaction and continuation, similar to rates of discontinuation seen in higher-income countries, 83–88% continuation rates were measured for LARC method users in the Contraceptive CHOICE project in the US [6]. The rates seen are also similar when compared to rates of discontinuation of other LARC methods in Africa where, in general, discontinuation is measured at 10% or lower for these methods across, with variability between countries (e.g., as high as 35% in Benin) and by method (e.g., up to 15% for the non-hormonal IUD) [7, 15]. The findings suggest that offering the hormonal IUD as part of a range of contraceptive options could benefit both clients who are looking for their first method of contraception and those who have tried other methods in the past. Additional factors, including the programmatic contexts in which these introductions took place highlighted below, may influence these results in a number of ways.

### Demographic profiles of users

The profiles of hormonal IUD users varied based on programmatic context, suggesting that the method can appeal to women regardless of their age, education, marital status, and parity. In two countries—Madagascar and Zambia—considerable proportions of those choosing the method were youth under age 25. The programs with the highest and lowest proportions of young clients, Madagascar and Nigeria respectively, were both private sector settings in which the hormonal IUD was offered at a higher price than other methods. A key difference between the Madagascar and Nigeria programs was that demand generation efforts in Madagascar prioritized reaching young professionals. The very low number of young users in Nigeria deserves further inquiry and may highlight a need for improved outreach to and counseling for this group. Finally, users across the two private sector settings tended to have high levels of education. Yet in Zambia, the only public sector service delivery context in this pilot, roughly half of hormonal IUD users had not completed secondary school. These findings highlight the potential for the hormonal IUD to meet the needs of diverse groups of women, especially when efforts are made to address equitable access by offering the method through multiple channels and service delivery models.

### Reasons for choosing the hormonal IUD, prior method use, and method switching

Having a range of contraceptive options available is important to meet the diverse needs of clients. The hormonal IUD appears to expand that range in a meaningful way by offering attributes that users did not see in other methods. In all pilot settings, prior method use was common, with the majority of women reporting that they had used other methods of contraception before choosing the hormonal IUD. Among women switching to the hormonal IUD who had been using a method recently, most had been using a short-acting method. In Madagascar and Zambia, the side effect profile unique to the hormonal IUD was among users’ top three reasons for selecting this method. No one method dominated as women’s preferred alternative to the hormonal IUD, had the hormonal IUD not been available. Notably, in Madagascar and Nigeria, many women (46% and 29%, respectively) reported that they would have left the facility with no method at all if unable to receive the hormonal IUD on the day of their service. As these were only quantitative surveys, it’s not possible to investigate exactly why women reported this, but further qualitative research could explore this question. If the hormonal IUD can meet the needs of women who do not see an acceptable alternative, or who would be dissatisfied with and discontinue an alternative method, the hormonal IUD has the potential to fill an important gap in the contraceptive market.

Reaching new users can help to reduce unmet need. Although most hormonal IUD users had tried other methods in the past, the study finds a large proportion of hormonal IUD clients in Zambia were new users. In Zambia, the hormonal IUD was offered in public sector facilities where all family planning methods were free. In the other two programs, the upfront cost to clients of the hormonal IUD service was higher than that of other methods. One could consider that in settings where price does not present an additional barrier when comparing method options, new users may be interested in a LARC method, like the hormonal IUD, as their first contraceptive method. Understanding the perceptions of new contraceptive users through additional research, in particular their experiences with bleeding changes and side effects, could help the community of practice to understand how methods like the hormonal IUD might be positioned to address the needs of new family planning clients.

### User satisfaction and continuation

As in high-income countries where the hormonal IUD is widely available, users in general appear to like this method. Satisfaction was high across all introductory countries, although it declined somewhat in Madagascar over time. Continuation rates at 12 months are high in all countries, though these rates should be interpreted with caution given high loss to follow-up from baseline through the periods of follow-up. Reasons for discontinuation over time can add more nuance to this picture. Common reasons for discontinuation among those no longer using the method at 3 months were often method related and include pain with the method and other side effects. Notably, in Madagascar and Nigeria, no user cited bleeding disturbances as a reason for discontinuation at 3 months whereas in Zambia, these were the most common reasons reported. Among users who had stopped using their method at 12 months, however, bleeding changes were the most common reason cited in all three countries.

With a closer look at discontinuation and satisfaction in Madagascar, where the bleeding profile was among the most common reasons for choosing the hormonal IUD, this could suggest a relationship between demand generation messages, the initial motivations for choosing the method, and the reasons for continuation over time. Hormonal IUD users are likely to experience changes to their bleeding throughout the 1st year following insertion, with amenorrhea more common in later months [16]. It is possible that users who were initially satisfied with the bleeding patterns they experienced may later have found bleeding changes like amenorrhea less appealing. This is conjecture, but it highlights the need for further qualitative research with users to explore the perception of bleeding changes throughout the period of method use. These findings also underscore the value of continued support for method users to manage and understand contraceptive induced bleeding changes.

Finally, though the sample of discontinuers is quite small, there were reports of expulsion in all three countries prior to 3 months. As this was not a clinical study this limited data may not provide a full picture of the expulsion rates among this cohort. However, this does have programmatic implications as training providers to mitigate the risk of expulsion during insertion and follow-up visits should be a priority to ensure quality care.

### Limitations

There were several limitations to consider. First, in each country this was a relatively small-scale pilot with a modest sample size powered on method satisfaction. Women who had selected the hormonal IUD as her method of choice were purposively selected for inclusion in the pilot study and this study was not intended to be a full evaluation of the hormonal IUD introduction in each country but rather to begin to build the evidence base for this method in LMIC markets. As the introduction of the hormonal IUD expands in LMICs, future research should consider designing similar, larger studies powered for this type of comparative analysis, either between countries/settings or between the hormonal IUD and other contraceptive methods.

Second, in two of the three countries, there was high loss to follow-up from baseline to the 3-month follow-up. The high loss to follow-up in Madagascar and Nigeria can be largely attributed to the follow-up mechanism of the surveys. Women were followed-up in these countries by mobile phone and many numbers were switched off when researchers attempted to follow-up and many calls went unanswered. In Zambia, loss to follow-up was much lower as researchers followed-up in person and were able to schedule follow-ups at times convenient for the participants. However, analysis did not find any evidence of differential attrition in any of the study countries based on demographics of women at baseline and at follow-up. We do not, however, know the discontinuation rate among those women that were lost to follow-up and whether it is similar, or not, to those that did participate in all follow-up surveys. Additionally, the baseline sample sizes in Madagascar and Nigeria were inflated midway through the study when higher loss to follow-up was experienced than anticipated, in an effort to ensure adequate power for analysis at 12-month follow-up. Because this is a pilot study of a method introduction, it is possible that this recruitment of women later in the study than initial recruitment introduced bias into the findings, particularly around continuation.

Third, in Madagascar and Nigeria, study participation was restricted to women who were able to provide a mobile phone number for follow-up. This likely biases our sample towards wealthier women who are more likely to own a phone. Analysis found no evidence of bias based on age when comparing age distribution within the study to the age distribution of routine Health Management Information Systems (HMIS) data recording uptake of the hormonal IUD, except in Nigeria where there was a higher proportion of young women who chose the hormonal IUD according to HMIS data than was represented in the study sample.

Finally, while the hormonal IUD was introduced into a range of markets through this work, including both the public and private sectors, these three markets were not representative of all LMIC markets, which are heterogenous.

Nevertheless, these largely descriptive results provide rich insights about hormonal IUD users in introductory settings where the method is on the cusp of scale-up.

## Conclusion

The introduction of the hormonal IUD could be impactful in global efforts to expand method choice. Increasing access to this method would give women in need a unique option that could both interest new users and provide an alternative for women who have been dissatisfied with and discontinued use of other methods. These introductory settings have shown that users across age and demographic segments would be interested in using the method and have high rates of satisfaction and continuation. Further evidence is needed to build upon these findings, such as to understand the relationship between user experience with the hormonal IUD and prior method use, as well as to understand the potential demand for the hormonal IUD at different comparative price points. As donors, organizations, and ministries of health consider expanding method offerings through hormonal IUD introductions, these findings suggest that the hormonal IUD could be well received and may contribute to greater uptake of modern, effective contraception.

## Data Availability

All data for this manuscript are available publicly in a de-identified format from USAID’s Data Development Library (DDL).

## Abbreviations

CI: Confidence interval
EECO: Expanding Effective Contraceptive Options
HMIS: Health Management Information Systems
ICA: International Contraceptive Access
IPC: Interpersonal Communication
IUD: Intrauterine Device
LARC: Long-Acting Reversible Contraception
LMIC: Low- and middle-income country
mCPR: Modern contraceptive prevalence rate
NHREC: National Health Research Ethics Committee
PSI: Population Services International
SFH: Society for Family Health
SIFPO2: Support for International Family Planning and Health Organizations 2
UNFPA: United Nations Population Fund
USAID: United States Agency for International Development
WCG: WCG Cares
